# Influence of germline variations in drug transporters ABCB1 and ABCG2 on intracerebral osimertinib efficacy in patients with non-small cell lung cancer

**DOI:** 10.1016/j.eclinm.2023.101955

**Published:** 2023-04-13

**Authors:** G.D. Marijn Veerman, Rene J. Boosman, Merel Jebbink, Esther Oomen-de Hoop, Anthonie J. van der Wekken, Idris Bahce, Lizza E.L. Hendriks, Sander Croes, Christi M.J. Steendam, Evert de Jonge, Stijn L.W. Koolen, Neeltje Steeghs, Ron H.N. van Schaik, Egbert F. Smit, Anne-Marie C. Dingemans, Alwin D.R. Huitema, Ron H.J. Mathijssen

**Affiliations:** a Department of Medical Oncology, Erasmus MC Cancer Institute, Erasmus University Medical Centre, Rotterdam, the Netherlands; b Department of Pulmonary Medicine, Erasmus MC Cancer Institute, Erasmus University Medical Center, Rotterdam, the Netherlands; c Department of Pharmacy and Pharmacology, The Netherlands Cancer Institute, Amsterdam, the Netherlands; d Department of Pulmonary Medicine, The Netherlands Cancer Institute, Amsterdam, the Netherlands; e Department of Pulmonary Medicine, University Medical Centre Groningen, University of Groningen, Groningen, the Netherlands; f Department of Pulmonary Medicine, Amsterdam University Medical Centres, Location Vrije Universiteit, Amsterdam, the Netherlands; g Department of Pulmonary Medicine, Maastricht University Medical Centre, GROW – School for Oncology and Reproduction, Maastricht, the Netherlands; h Department of Clinical Pharmacy & Toxicology, Maastricht University Medical Centre, CARIM – School for Cardiovascular Disease, Maastricht, the Netherlands; i Department of Pulmonary Medicine, Amphia Hospital, Breda, the Netherlands; j Department of Clinical Chemistry, Erasmus University Medical Centre, Rotterdam, the Netherlands; k Department of Hospital Pharmacy, Erasmus University Medical Centre, Rotterdam, the Netherlands; l Department of Medical Oncology and Clinical Pharmacology, The Netherlands Cancer Institute, Amsterdam, the Netherlands; m Department of Pulmonary Medicine, Leiden University Hospital, Leiden, the Netherlands; n Department of Pharmacology, Princess Maxima Center for Paediatric Oncology, Utrecht, the Netherlands; o Department of Clinical Pharmacy, University Medical Center Utrecht, Utrecht University, Utrecht, the Netherlands

**Keywords:** Osimertinib, Brain metastases, Drug transporters, Single-nucleotide polymorphism, Pharmacokinetics, Non-small cell lung cancer

## Abstract

**Background:**

Central nervous system (CNS) metastases are present in approximately 40% of patients with metastatic epidermal growth factor receptor-mutated (*EGFR*m+) non-small cell lung cancer (NSCLC). The EGFR-tyrosine kinase inhibitor osimertinib is a substrate of transporters ABCB1 and ABCG2 and metabolized by CYP3A4. We investigated relationships between single nucleotide polymorphisms (SNPs) *ABCB1* 3435C>T, *ABCG2* 421C>A and 34G>A, and *CYP3A4∗22* and CNS treatment efficacy of osimertinib in *EGFR*m+ NSCLC patients.

**Methods:**

Patients who started treatment with osimertinib for *EGFR*m+ NSCLC between November 2014 and June 2021 were included in this retrospective observational multicentre cohort study. For patients with baseline CNS metastases, the primary endpoint was CNS progression-free survival (CNS-PFS; time from osimertinib start until CNS disease progression or death). For patients with no or unknown baseline CNS metastases, the primary endpoint was CNS disease-free survival (CNS-DFS; time from osimertinib start until occurrence of new CNS metastases). Relationships between SNPs and baseline characteristics with CNS-PFS and CNS-DFS were studied with competing-risks survival analysis. Secondary endpoints were relationships between SNPs and PFS, overall survival, severe toxicity, and osimertinib pharmacokinetics.

**Findings:**

From 572 included patients, 201 had baseline CNS metastases. No SNP was associated with CNS-PFS. Genotype *ABCG2* 34GA/AA and/or *ABCB1* 3435CC --present in 35% of patients-- was significantly associated with decreased CNS-DFS (hazard ratio 0.28; 95% CI 0.11–0.73; p = 0.009) in the multivariate analysis. This remained significant after applying a Bonferroni correction and internal validation through bootstrapping. *ABCG2* 421CA/AA was related to more severe toxicity (27.0% versus 16.5%; p = 0.010).

**Interpretation:**

ABCG2 34G>A and *ABCB1* 3435C>T are predictors for developing new CNS metastases during osimertinib treatment, probably because of diminished drug levels in the CNS. *ABCG2* 421C>A was significantly related with the incidence of severe toxicity. Pre-emptive genotyping for these SNPs could individualize osimertinib therapy. Addition of ABCG2 inhibitors for patients without *ABCG2* 34G>A should be studied further, to prevent new CNS metastases during osimertinib treatment.

**Funding:**

No funding was received for this trial.

**Research in context**

**Evidence before this study**

We performed a structured search in PubMed and Embase for preclinical and clinical studies until November 21, 2022, written in English, with the search terms “Osimertinib AND (ABCB1 OR ABCG2 OR CYP3A4)”. This resulted in 16 and 65 records respectively. *In vitro* research showed that osimertinib is a substrate of the efflux transporters ABCB1 and ABCG2. This was further confirmed in mice. Intracerebral osimertinib accumulation was limited by both transporters, which was demonstrated in ABCB1/ABCG2 knockout mice which had six-fold higher osimertinib exposure in the brain. Osimertinib is a substrate of CYP3A4 *in vitro* and *in vivo*. When CYP3A was knocked-out in mice however, no difference in osimertinib pharmacokinetics was observed. Nevertheless, in humans with the *CYP3A4∗22* variant allele, the exposure to other substrate drugs (including some tyrosine kinase inhibitors) was increased. For osimertinib, this has not been studied yet. Single nucleotide polymorphisms (SNPs) in *ABCB1*, *ABCG2*, or *CYP3A4* have not been correlated with clinical endpoints before in patients who are treated with osimertinib.

**Added value of this study**

This is the first study to correlate SNPs in relevant efflux transporters to osimertinib effectiveness and severe toxicity in patients with non-small cell lung cancer (NSCLC). More specifically, SNPs *ABCB1* 3435C>T and/or *ABCG2* 34G>A were significantly correlated with the development of central nervous system (CNS) metastases in patients without CNS metastases at the start of osimertinib treatment. Furthermore, *ABCG2* 421C>A was significantly related with the incidence of severe toxicity.

**Implications of all the available evidence**

Patients with CNS metastases have a lowered overall survival and quality of life compared to patients without CNS metastases. Using the predictive SNPs in *ABCB1* and/or *ABCG2*, patients can be identified who are at higher risk of developing CNS metastases during osimertinib treatment. Osimertinib therapy could be individualized when pre-treatment genotyping is implemented after prospective validation of these results. Ultimately, ABCB1 and ABCG2 could function as promising therapeutic target in future trials to prevent the development of new CNS metastases.

## Introduction

Central nervous system (CNS) metastases are present in almost 40% of patients with metastatic non-small cell lung cancer (NSCLC).^1^^,^^2^ In patients with an epidermal growth factor receptor mutation (*EGFR*m+), this number is similar or slightly higher.^2^^,^^3^ The cumulative incidence of CNS metastases only rises further during treatment.^4^^,^^5^ As a consequence, the presence of CNS metastases negatively affects both overall survival (OS) and quality of life.^1^^,^^2^

Systemic treatment of brain metastases is often complicated by the limited drug penetration across the blood–brain barrier. Its physiologic properties prevent large molecules to cross, and efflux transporters --predominantly ABCB1 and ABCG2-- actively transport molecules (back) into the blood, resulting in pharmacologic failure and intracerebral drug resistance.^6^^,^^7^ Therefore, the influence of ABCB1 and ABCG2 on intracerebral drug accumulation has been studied intensively. It was found that drugs which are substrates of both transporters accumulate vastly when ABCB1 and ABCG2 are dysfunctional.^7^ For example, in primates, the intracerebral accumulation of the first-generation EGFR-tyrosine kinase inhibitor (TKI) erlotinib increased 5-fold when concomitantly treated with a dual ABCB1/ABCG2 inhibitor.^8^

The third generation EGFR-TKI osimertinib has the highest blood–brain barrier penetration --and hence intracerebral drug accumulation-- compared with first and second generation EGFR-TKIs.^9^ The incidence of CNS disease progression --including *de novo* occurrence of brain metastases-- was significantly lower in patients treated with osimertinib compared to patients treated with erlotinib and gefitinib (23% versus 44% after 12 months).^4^ These results further confirmed the superiority of osimertinib over first-generation EGFR-TKIs as first-line treatment for *EGFR*m+ advanced NSCLC.^10^

Osimertinib and its active metabolites are both substrates of ABCB1 and ABCG2.^11^ When these transporters were knocked-out in mice, this resulted in a 6-fold higher intracerebral drug accumulation.^12^ In clinical practice, common genetic variants are known to significantly impair the function of ABCB1 and ABCG2. These single nucleotide polymorphisms (SNPs) increase the systemic drug exposure, and subsequently increase survival and toxicity of several other EGFR-TKIs.^11^ The influence of these SNPs on the (intracerebral) efficacy of osimertinib has not been studied *in vivo* yet. Most frequently described are the 3435C>T SNP in *ABCB1* (prevalence CC 26–31%, CT 44–47%, TT 22–30%) and the 421C>A and 34G>A SNPs in *ABCG2* (prevalence CC 74–86%, CA 14–26%, AA <1% and GG 91%, AG 9%, AA <1% respectively).^11^ All three variants may lead to decreased drug transport through reduced protein expression and/or function^13^, ^14^, ^15^ and have been associated with survival and toxicity in clinical studies.^11^ The X>Z notation describes the germline variant which can occur at a specific nucleotide position. There are three possible nucleotide combinations, in which XX means (homozygote) wild-type, XZ means heterozygote variant, and ZZ means homozygote variant.

Furthermore, the effects of genetic variations in ABCB1 and ABGCG2 on systemic osimertinib concentrations are unknown. Osimertinib is mainly metabolized by cytochrome P450 isoenzyme 3A4 (CYP3A4).^11^ The CYP3A4∗22 variant allele (prevalence 10%) is associated with low gastro-intestinal and hepatic CYP3A4 expression and thus low activity,^16^ potentially increasing the systemic --and subsequent intracerebral-- concentrations of osimertinib.

**Fig. 1:**
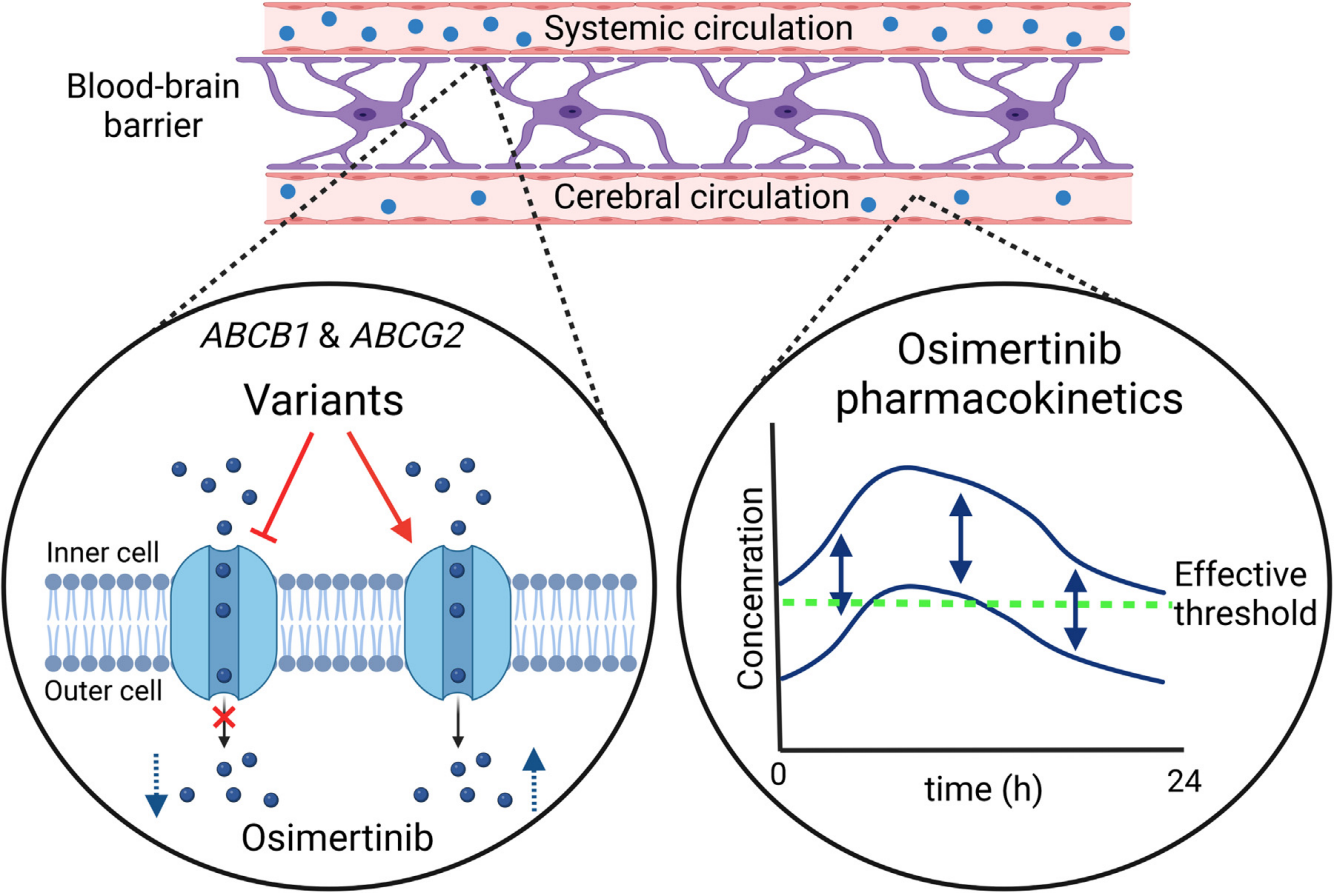
**Possible effects of single nucleotide polymorphisms in ABCB1 and ABCG2 on the osimertinib concentration in the central nervous system.** The blue spheres represent osimertinib. The red arrows in the left circle show the possible decreased (--|) or normal (-->) function of the osimertinib transporting enzymes ABCB1 and ABCG2, which could lead to altered osimertinib concentrations in the central nervous system (right circle).

For this study, we hypothesized that SNPs in *ABCB1*, *ABCG2*, and *CYP3A4* could influence CNS efficacy of osimertinib, *c.q.* intracerebral progression-free survival (CNS-PFS) and occurrence of new CNS metastases, as shown in Fig. 1. Furthermore, the influence of these SNPs on PFS and OS, severe toxicity and osimertinib pharmacokinetics was studied.

## Methods

### Study design & patient selection

This is a retrospective, cross-sectional observational cohort study, performed in six large cancer centres in the Netherlands. Patients were eligible for inclusion if they were ≥18 years and treated with osimertinib for *EGFR*m+ NSCLC between November 2014 and June 2021. Patients were excluded when no plasma or whole blood for SNP analysis was available. Data cut-off was January 2022 to ensure a minimal follow-up time of 6 months. Fig. S1 presents a flowchart to account for the origin of the collected data. Prior systemic treatment was allowed, including chemotherapy, immunotherapy, and first/second generation EGFR-TKIs. Osimertinib had to be used as standard-of-care in a once-daily dosing schedule. Furthermore, whole blood or plasma had to be available for genotyping of the four germline variants *ABCB1* 3435C>T, *ABCG2* 421C>A and 34G>A, and *CYP3A4∗22*. To ensure single-blinding of the investigators, all clinical data were collected before any genotyping took place.

### Study procedures

Baseline imaging was performed according to local practice, which was mostly within 4 weeks prior to start of osimertinib treatment. Imaging of the brain was performed according to local practice, and was mainly performed in patients with known CNS metastases or symptoms suggestive of CNS metastases. Regular imaging of the brain was not performed. Dose modifications, interruptions and discontinuation due to toxicity and the frequency of radiologic treatment evaluation were at the treating physicians’ discretion. Normally, the first radiological evaluation took place with a computed-tomography (CT)-scan after four to eight weeks, and thereafter once every 8–12 weeks until disease progression. Central nervous system metastases were followed-up with magnetic resonance imaging (MRI). Baseline characteristics and clinical data were recorded and anonymised. Since the variants of interest are germline SNPs, the timing of blood withdrawal for genotyping was not relevant. For pharmacokinetic evaluation, the time between last osimertinib administration and blood withdrawal had to be known. Only plasma concentrations at the time of steady state (*c.q.* after 2 weeks) at a dosage of 80 mg once daily were used for comparison. Furthermore, since osimertinib is unstable in whole blood and plasma at room temperature,^17^ pharmacokinetic samples were stored on ice and worked-up for quantification within 1 h. Since the time of maximum concentration of osimertinib is 6 h, only samples taken six or more hours after drug administration were eligible for analysis. These data also included pre-collected samples from earlier studies (Fig. S1).^18^^,^^19^

DNA was isolated from whole blood or plasma with the MaxWell kit (Promega, AS1520). The digital droplet PCR analyses were performed in the T100 Thermal Cycler and detected with the QX200 Droplet Reader. QuantaSoft (BioRad) was used for DNA quantification and subsequent genotyping the *ABCB1* 3435C>T, *ABCG2* 421C>A and 34G>A, and *CYP3A4∗22* variants.

For the primary analyses, patients were divided in two cohorts, depending on the presence of CNS metastases at start of osimertinib treatment. CNS metastases were present at baseline when proven by MRI, CT-scan or pathologic diagnostics of cerebral spinal fluid --in case of leptomeningeal metastases-- prior to start of osimertinib treatment. Baseline CNS metastases were only defined as absent if these were not present on MRI, since with (contrast-enhanced) CT scanning micro-metastases and leptomeningeal metastases could be missed. All other patients had unknown CNS metastases at baseline.

### Study endpoints

In the cohort with known CNS metastases at baseline, primary outcome was the relationship between the four SNPs with CNS-PFS, defined as the time from treatment start until progressive CNS disease according to the Response Assessment in Neuro-Oncology Brain Metastases (RANO-BM) criteria^20^ or death from any cause. In the cohort without or with unknown CNS metastases at baseline, primary endpoint was the relation between the four SNPs and time to the *de novo* occurrence of CNS metastases (CNS disease-free survival; CNS-DFS).

Secondary endpoints were the relationships between presence of the four SNPs and OS (*i.e.*, time from treatment start until death from any cause), PFS independent of disease site, severe toxicity, and osimertinib pharmacokinetics (mean minimal plasma concentration; C_min_) in the total cohort. In the PFS analysis, an event was disease progression according to the Response Evaluation Criteria in Solid Tumors (RECIST) version 1.1,^21^ or if it was treated as such --with radiotherapy for oligometastatic disease or change of treatment-- by the treating physician, or death from any cause occurred. Severe toxicity was scored as Common Terminology Criteria for Adverse Events (CTCAE)^22^ grade >2 adverse events, and all toxicity which led to dose reductions, treatment discontinuation or stop, or hospital admissions.

### Statistical analysis

For the primary endpoints, CNS-PFS and CNS-DFS were related with every SNP and patient characteristics as single parameter using univariate proportional hazards models for the subdistribution (results denoted by the subdistributional hazard ratio [sHR]), as described by Fine and Gray.^23^ Parameters with a p-value <0.200 in the univariate analysis were entered in a multivariable proportional hazards model for the subdistribution where backward selection was applied with p < 0.05 as cut-off for statistical significance. Competing-risks analysis prevents overestimation of the incidence of the events of interest.^24^ For the CNS-PFS analysis, competing risks were defined as other events than CNS-progression or death which caused osimertinib treatment to stop (*c.q.* severe toxicity or change of therapy due to extracerebral progressive disease). For the CNS-DFS analysis, death from any cause was also considered to be a competing risk additional to other reasons for treatment stop. Internal validation of all SNPs and statistically significant parameters in the multivariable analyses of the primary endpoints was performed by bootstrapping 1000 samples.^25^ To correct for the use of four unique SNPs, the Bonferroni method to adjust for multiplicity was applied.^26^ Herewith, only SNPs with p < 0.0125 were considered statistically significant.

For the secondary endpoints OS and PFS, every SNP and patient characteristics were first tested as single parameter with the Kaplan–Meier method and log-rank test. Thereafter, Cox proportional-hazards regression analysis was used for parameters with a p-value <0.10. With the chi-square test and competing risk analyses,^23^ differences in the incidence of severe toxicity and time-to-severe toxicity were analysed respectively. Parameters with a p < 0.200 in the univariate analysis were entered in a multivariable model, in which p < 0.05 was statistically significant. Osimertinib C_min_ was calculated with the earlier published methods.^18^^,^^19^ These mean trough concentrations were compared with the four SNPs using the non-parametric Mann–Whitney test, since C_min_ is considered to be not-normally distributed.

Germline variants with minor allele frequencies ≤0.10 were only tested in a dominant model (*c.q.* wild-type versus heterozygote and homozygote variants). The other variants were tested in both dominant and recessive (*c.q.* wild-type and heterozygote variants versus homozygote variants) models. If single SNPs had p < 0.20 in the primary univariate competing risk analysis, combined testing of two SNPs was considered acceptable.

All statistical tests were performed using version 28.0.1 SPSS for Windows (Statistical Package for Social Sciences, Chicago, IL) or Stata (StataCorp. 2019. Stata: Release 16.1. Statistical Software. College Station, TX: StataCorp LP), and p < 0.05 was considered to be significant unless stated otherwise.

### Ethics approval

The study was primarily approved by the local ethics committee (Erasmus University Medical Center Rotterdam; MEC 20-557) and was registered in the Dutch Trial Registry and International Clinical Trial Registry Platform (www.trialsearch.who.int; trial NL8914). Thereafter, the local Medical Ethics Committee at every participating site approved the study protocol independently. Since this study was observational, non-invasive, and already collected plasma samples were used, all Medical Ethics Committees waived the obligation for written informed consent of patients. Clinical data and patients samples were obtained after inclusion in local trials: START-TKI Rotterdam and Breda (MEC 16-643, www.clinicaltrials.gov NCT05221372), N13FPB The Netherlands Cancer Institute (IRBd19-192), Maastricht 2019-1018-A-10 and Maastricht OSIBOOST (www.clinicaltrials.gov NCT03858491). Patients from Groningen UMC were included from the Oncolifes biobank, from which the request was approved by the local ethics committee (OLS032-202000693). Patients from Amsterdam UMC were included from the Liquid Biopsy Center biobank, from which the request was approved by the local institutional review board (UVB21-0125). Flow chart for patient samples and data, including ethical approval, is presented in Fig. S1.

### Role of the funding source

No funding was received in any form, for the execution of this study. There are no conflicts of interest nor funding for the collection, analysis, and interpretation of data, in the writing of the report, nor in the decision to submit the paper for publication.

## Results

A total of 572 patients with *EGFR*m+ NSCLC were included. Median time of follow-up was 27.7 months. Baseline characteristics are depicted in Table 1. The distributions in sex and age were comparable compared to earlier research, as well as primary EGFR-mutation status and frequency of prior cranial radiotherapy.^4^ In this cohort, relatively more Caucasian patients have been included. CNS metastases were present at baseline in 201 (35%) patients. Osimertinib was used as first line treatment in 169 (30%) patients, and was not correlated with presence of CNS metastases at baseline (Chi-square p = 0.272). In 82% of the patients with CNS metastases, the CNS metastases could be used as measurable or evaluable disease. Genotyping was successful in ≥99.5% of all included patients. Table 2 shows the prevalence and minor allele frequency of the genotyped SNPs. The prevalence of every SNP was similar to historic data^11^^,^^13^ and in Hardy–Weinberg equilibrium (p > 0.05).

**Table 1:** Patient demographics.

Demographic	Total cohort (n = 572)	Patients with known CNS metastases (n = 201)	Patients without known CNS metastases (n = 371)
Sex			
Male	176 (31%)	52 (26%)	124 (33%)
Female	396 (69%)	149 (74%)	247 (67%)
Race			
Caucasian	518 (91%)	175 (87%)	343 (93%)
Asian	40 (7%)	18 (9%)	22 (6%)
Other	14 (2%)	8 (4%)	6 (2%)
Age (years) median [IQR]	65.7 [58.2–72.8]	63.5 [56.4–70.1]	67.5 [59.3–74.2]
Body mass index (kg/m^2^) median [IQR]	24.2 [21.9–27.2]	24.1 [21.6–27.1]	24.4 [22.1–27.3]
WHO performance status at baseline			
0	157 (27%)	45 (22%)	112 (30%)
1	318 (56%)	112 (56%)	206 (56%)
2	78 (14%)	33 (16%)	45 (12%)
3	17 (3%)	9 (5%)	8 (2%)
4	2 (0.3%)	2 (1%)	–
Smoking status			
Never	280 (49%)	96 (48%)	184 (50%)
Former	272 (48%)	97 (48%)	175 (47%)
Current	20 (4%)	8 (4%)	12 (3%)
Primary EGFR mutation^a^			
Classic exon 19 deletion	333 (58%)	109 (54%)	224 (61%)
Exon 21 L858R	163 (29%)	61 (30%)	102 (28%)
Other (single or compound mutations)	75 (13%)	31 (15%)	44 (12%)
Presence of T790M mutation at baseline			
Yes	343 (60%)	102 (51%)	242 (65%)
No	229 (40%)	99 (49%)	130 (35%)
Presence of TP53 mutation at baseline			
Yes	201 (35%)	93 (31%)	168 (45%)
No	261 (46%)	62 (46%)	139 (38%)
Unknown	110 (19%)	46 (23%)	64 (17%)
Treatment line			
First	169 (30%)	51 (25%)	118 (32%)
Second	403 (70%)	150 (75%)	253 (68%)
Prior non-EGFR targeted therapy^b^			
None	444 (78%)	148 (74%)	296 (80%)
Chemotherapy^c^	88 (15%)	35 (18%)	53 (14%)
Chemotherapy + radiotherapy	31 (5%)	13 (7%)	18 (5%)
Chemotherapy + immunotherapy	8 (1%)	4 (2%)	4 (1%)
Presence of brain metastases at baseline			
Yes	201 (35%)	201 (100%)	–
No	96 (17%)	–	96 (26%)
Unknown	275 (48%)	–	275 (74%)
Cranial radiotherapy^b^			
No	488 (85%)	117 (58%)	371 (100%)
Yes – stereotactic radiotherapy	46 (8%)	46 (23%)	–
Yes – whole brain radiotherapy	37 (7%)	37 (18%)	–
Time between last day of radiotherapy and start of osimertinib treatment (days) median [IQR]	255 [96–544]	255 [96–544]	–

Abbreviations: n = number of patients; IQR = interquartile range; kg = kilograms; m = meters; WHO = World Health Organisation; EGFR = epidermal growth factor receptor; QD = once daily.

a One patient with unknown data in the cohort without known CNS metastases.

b One patient with unknown data in the cohort with known CNS metastases.

c Two patients received both chemotherapy and a tyrosine kinase inhibitor sequentially.

**Table 2:** Prevalence of genotyped single nucleotide polymorphisms.

Gene	Single nucleotide polymorphism	International reference number	Patients successfully genotyped (total n = 572)	Homozygous wild-type	Heterozygous	Heterozygous wild-type	Minor allele frequency
*ABCB1*	3435C>T	rs1045642	572	137	282	153	0.514
*ABCG2*	421C>A	rs2231142	569	454	112	3	0.104
*ABCG2*	34G>A	rs2231137	569	486	72	11	0.083
*CYP3A4*	15389C>T (∗22)	rs35599367	570	505	64	1	0.058

The results of the primary analyses are presented in Table 3. In the cohort with CNS metastases at start of osimertinib treatment, 108 patients (54%) experienced a CNS-PFS event after a median of 19.4 months (cumulative incidences of 41.0% and 54.8% after 12 and 24 months respectively). In the univariate analyses, no SNP was significantly associated with CNS disease progression, nor was any SNP eligible to be included in the multivariable analysis (all p > 0.20). Parameters which were associated with improved CNS-PFS were primary *EGFR*-mutation (favouring the classic exon 19 deletion), presence of the T790M mutation, and not having received prior chemo- and/or immunotherapy (Table 3).

**Table 3:** Univariate and multivariate analyses of patient demographics and SNPs associated with CNS-PFS and CNS-DFS.

Parameter	Patients with CNS metastases at baseline (n = 201), CNS-PFS			Patients without CNS metastases at baseline (n = 371), CNS-DFS		
	Univariate competing risk HR (95% CI; p-value)	Multivariate competing risk HR (95% CI)	Validation^a^ 95% CI	Univariate competing risk HR (95% CI; p-value)	Multivariate competing risk HR (95% CI)	Validation^a^ 95% CI
Sex						
Female vs male	0.939 (0.612–1.442; 0.775)			0.967 (0.484–1.931; 0.924)		
Age (per year)	0.988 (0.973–1.004; 0.147)			0.984 (0.959–1.009; 0.201)		
Ethnicity						
Asian vs other	0.652 (0.340–1.247; 0.196)			0.438 (0.061–3.144; 0.411)		
BMI (in kg/m^2^)	1.031 (0.984–1.080; 0.205)			0.962 (0.907–1.019; 0.189)		
WHO PS						
>1 vs 0–1	1.019 (0.637–1.630; 0.936)			2.166 (1.022–4.590; 0.044)	2.208 (1.004–4.853^b^)	0.852–4.727
Smoking						
Former/current vs never	1.364 (0.933–1.994; 0.109)			1.289 (0.669–2.484; 0.448)		
Primary EGFR mutation^c^						
pL858R vs classic exon 19 del	2.165 (1.435–3.265; 0.000)	2.059 (1.343–3.159^b^)	1.391–3.154	2.904 (1.408–5.991; 0.004)	2.832 (1.372–5.848^b^)	1.282–6.364
Other vs classic exon 19 del	2.554 (1.470–4.436; 0.001)	2.320 (1.331–4.046^b^)	1.261–4.005	3.061 (1.215–7.713; 0.018)	2.785 (1.071–7.238^b^)	0.931–7.428
Presence of T790M						
Yes vs no	0.526 (0.358–0.772; 0.001)	0.595 (0.399–0.889^b^)	0.396–0.881	1.175 (0.569–2.425; 0.662)		
Presence of TP53						
Yes vs no	1.262 (0.797–1.999; 0.321)			1.557 (0.772–3.138; 0.216)		
Line of treatment						
Second vs first	1.241 (0.790–1.950; 0.349)			1.363 (0.622–2.987; 0.439)		
Other prior treatment^d^						
Yes vs no	1.406 (0.920–2.151; 0.116)	1.545 (1.017–2.348^b^)	1.019–2.334	1.475 (0.706–3.081; 0.301)		
Cranial radiotherapy^d^						
Yes vs no	0.789 (0.535–1.163; 0.231)			–		
*ABCB1* 3435C>T dominant						
CT/TT vs CC	1.279 (0.799–2.047; 0.305)		0.804–2.225	2.667 (0.949–7.489; 0.063)		1.168–11.360
*ABCB1* 3435C>T recessive						
TT vs CT/CC	1.274 (0.861–1.885; 0.225)		0.841–1.857	1.795 (0.915–3.523; 0.089)		0.903–3.456
*ABCG2* 421C>A dominant						
CA/AA vs CC	1.269 (0.798–2.017; 0.313)^e^		0.793–2.063	1.940 (0.976–3.857; 0.059)^g^		0.906–3.807
*ABCG2* 34G>A dominant						
GA/AA vs GG	1.011 (0.597–1.710; 0.969)^e^		0.576–1.683	0.156 (0.021–1.153; 0.069)^g^	NA	0.000–0.559
*CYP3A4∗22* dominant						
CT/TT vs CC	1.383 (0.691–2.769; 0.360)^f^		0.598–2.799	0.394 (0.095–1.643; 0.201)^g^		0.000–1.170
Combined genotype						
*ABCB1* 3435 CC and/or *ABCG2* 34 GA/AA vs *ABCB1* 3435 CT/TT and *ABCG2* 34 GG	0.887 (0.597–1.317; 0.553)		0.568–1.271	0.281 (0.110–0.721; 0.008)	0.282 (0.110–0.725^b^)	0.078–0.669

Association between patient demographics and SNPs with progression-free survival (CNS-PFS) and disease-free survival (CNS-DFS) in the central nervous system.

Abbreviations: SNP = single nucleotide polymorphism; CNS = central nervous system; HR = hazard ratio; CI = confidence interval; BMI = body mass index; kg = kilograms; m = meter; WHO = World Health Organisation; EGFR = epidermal growth factor receptor; vs = versus; NA = not available, because the number of events in the GA/AA cohort was too little.

a Validation was reported for multivariate analyses for parameters which were statistically significant. Only for other SNPs, univariate validation was reported.

b p < 0.05.

c One patient with unknown data in the cohort without known CNS metastases.

d One patient with unknown data in the cohort with known CNS metastases.

e In 199 patients.

f In 200 patients.

g In 370 patients.

**Fig. 2:**
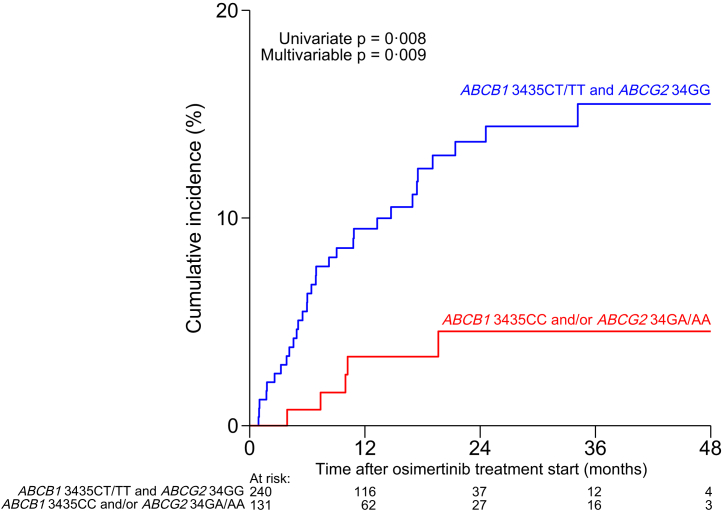
**Central nervous systemefficacy of osimertinib in patients without known brain metastases.** Cumulative incidence of central nervous system metastases in patients which are divided based on the combined genotype *ABCB1* 3435C>T - *ABCG2* 34G>A. The blue line represents patients with *ABCB1* 3435CC and/or *ABCG2* 34GA/AA (combined genotype +), the red line represents patients with *ABCB1* 3435CT/TT and *ABCG2* 34GG (combined genotype -). The 12-months incidence for the combined genotype + cohort was 3.3%, compared to 9.5% in the combined genotype - cohort.

In the cohort without known CNS metastases at start of osimertinib treatment, 36 patients (9.7%) experienced a CNS-DFS event, with cumulative incidences of 7.2% and 10.4% after 12 and 24 months respectively. All SNPs except the *CYP3A4*∗22 variant allele had univariate p < 0.20, as shown in Table 3. However, because there was only one single CNS-DFS event (1.8%) in 55 patients with an *ABCG2* 34G>A SNP, compared to 35 events (11.1%) in 315 wild-type patients, it was statistically impossible to incorporate this SNP in the multivariable analysis as this gave estimation problems. In order to be able to use this *ABCG2* 34G>A SNP (Fig. S2A), we analysed it in combination with *ABCB1* 3435C>T (Fig. S2B). We choose not to combine the two *ABCG2* SNPs to avoid possible linkage disequilibrium in the same gene. With the combined genotype, 131 patients with *ABCB1* 3435CC and/or *ABCG2* 34GA/AA were compared to 240 patients with *ABCB1* 3435CT/TT and *ABCG2* 34GG (Fig. 2). In the multivariable analysis this resulted in a significant HR of 0.282 (95% confidence interval (CI) 0.110–0.725; p = 0.009). This result remained statistically significant in the validation (95% CI 0.078–0.669) as well as when the Bonferroni correction was considered. *ABCG2* 421C>A did negatively affect CNS-DFS, but was excluded in the backward selection of the multivariable analysis (0.10 > p > 0.05). Other parameters, which were statistically significantly associated with reduced number of new occurrence of CNS metastases, were *EGFR* exon 19 as primary *EGFR* mutation and WHO performance status 0–1 at baseline (Table 3). Only the detrimental effect of the *EGFR* p.L858R compared to the classic exon 19 deletion (HR 2.832; 95% CI 1.372–5.848; p = 0.005) remained statistically significant after validation. Since in the AURA3 study^27^ only patients with T790M mutations were treated with osimertinib in the second line, we also analysed the cohort without the patients who did not meet this criterion (n = 15). This did not result in a different outcome.

In 572 patients, 455 (80%) experienced progression of disease with a median PFS of 10.2 months [IQR 8.9–11.5]. Two hundred eighty-eight (50%) patients died during follow-up, with a median OS of 25.3 months [IQR 23.0–27.5]. No SNPs were statistically significantly related with either PFS or OS (Table S1). Asian race, higher BMI, WHO performance status 0–1, classic *EGFR* exon 19 deletion and osimertinib used as first line were associated with improved PFS and OS. The presence of TP53 at baseline and known CNS metastases at baseline were associated with significantly decreased PFS and OS. Female sex and older age were significantly associated with improved PFS, but not with OS.

The incidence of severe osimertinib toxicity was significantly higher in patients with *ABCG2* 421CA/AA compared with wild-type patients (27.0% versus 16.5%; p = 0.010). In the univariate time-to-event analysis, the same *ABCG2* 421C>T SNP was significantly associated with higher hazard of severe toxicity (HR 1.687; 95% CI 1.115–2.554; p = 0.013; Fig. S3). However, it was removed from the multivariable model with the backward selection, with p = 0.062. Other parameters associated with more severe toxicity were female sex, older age and osimertinib used as first line of treatment. The results for all parameters are shown in Table S2.

*ABCB1* 3435C>T was associated with significantly decreased osimertinib plasma concentrations. In homozygote variant patients (TT), osimertinib C_min_ was 11% lower compared to CT/CC patients. No other SNP was associated with osimertinib pharmacokinetics (Table S2).

## Discussion

In this multicentre trial to study SNPs in important transporter genes, the combined genotype of *ABCG2* 34GA/AA and *ABCB1* 3435CC was highly predictive for decreased occurrence of new CNS metastases in patients without known CNS metastases when starting osimertinib treatment. These SNPs probably cause the intracerebral osimertinib concentration to increase through diminished osimertinib efflux across the blood–brain barrier. This result remained statistically significant after internal validation and applying a Bonferroni correction, and are regardless of treatment line. Implementation of these SNPs into clinical practise could help to guide diagnostic and therapeutic decision making (discussed in more detail below), and to offer a specific target for future research to prevent occurrence of new CNS metastases.

With the introduction of osimertinib, the prevalence of CNS disease progression and occurrence of new CNS metastases is lower compared to other EGFR-TKIs.^4^ However, there is still a considerable number of patients who have (solely) progressive CNS disease, which limits survival and quality of life.^1^^,^^2^ We identified patients with the genotype *ABCG2* 34GA/AA and/or *ABCB1* 3435CC (35.3% of the total cohort) to have a 72% reduced risk of developing new CNS metastases compared to patients with the *ABCG2* 34GG and *ABCB1* 3435CT/TT genotype. This large effect was predominantly because of the *ABCG2* 34G>A SNP (Table 3 and Fig. S2A), that probably reduced osimertinib efflux through the blood–brain barrier and causes the intracerebral osimertinib concentration to increase (Fig. 1) as was found in mice by Van Hoppe et al.^12^ This is the first report of ABCG2 being clinically important in the CNS efficacy of osimertinib, and offers a possible therapeutic target in patients without this SNP. Concomitant use of ABCG2 inhibitors (*e.g.*, anti-HIV protease inhibitors or dietary flavonoids^28^) is a potentially promising intervention to prevent the occurrence of new CNS metastases, and should be studied further. The clinical relevance of pre-emptive genotyping these SNPs to date, could be to guide monitoring strategies aimed at early detection of CNS metastases in the group of patients without the combined genotype. Early detection offers the benefit of being able to treat CNS metastases in the pre-symptomatic window.

In patients with known CNS metastases at baseline, not a single SNP was associated with CNS efficacy. This might be explained by the compromising effects of CNS metastases on the blood–brain barrier.^29^ When the blood–brain barrier loses its permeability function, various molecules (including osimertinib) can leak more easily across it.^29^ Hence, efflux transporters are not the limiting factor of intracerebral drug accumulation anymore. Another reason could be that a considerable number of patients had had cranial radiotherapy some time before osimertinib treatment started. Radiotherapy could have comparable detrimental effects on the integrity of the blood–brain barrier,^30^ although it was not associated with altered CNS-PFS (Table 3). As a consequence, the additional value of transporter proteins is probably limited, but more detailed research to the duration and intensity of radiotherapy might be necessary.

No SNP was associated with PFS or OS. This was expected, since there is recent literature that confirms an absent systemic exposure-efficacy relationship for osimertinib in the standard dosage.^18^^,^^19^

*ABCG2* 421C>A was significantly related with the incidence of severe osimertinib toxicity. The 20% of patients with this SNP experienced 64% more severe toxicity compared to wild-type patients. This clinically relevant effect could not be explained by higher systemic osimertinib concentrations (Table S1). These concentrations may however not represent the drug concentrations in specific compartments, which could be altered at tissue-level. Genotyping this SNP in future clinical practice would be relevant, since it could identify patients at risk for severe toxicity, and additional monitoring could be considered. Further research should focus on the mechanism behind this effect in order to advise on the possible therapeutic consequences. Moreover, the increased incidence in severe toxicity could explain the detrimental effect of *ABCG2* 421C>A on the occurrence of new CNS metastases. Although this effect was not statistically significant (probably because of a lack of power), it makes sense that more severe toxicity leads to more dose interruptions and reductions, which (temporarily) decrease the intracerebral osimertinib concentrations and hence increase the chance of CNS metastases.

Osimertinib trough levels were significantly lower in patients with an *ABCB1* 3435C>T SNP. This could be caused by the impaired drug transport across the basolateral intestinal cell membrane into the systemic circulation, that results in lower drug absorption.^11^ This may thus also be a reason why the *ABCB1* 3435CC genotype contributes to the reduced risk in developing new CNS metastases. However for PFS, OS, or the incidence of severe toxicity, an 11% decrease in C_min_ will probably not be clinically relevant, especially since no systemic exposure-efficacy relationship for osimertinib has been shown.^18^^,^^19^ However, an exposure-efficacy relationship for the CNS has not been studied yet.

The baseline characteristics which are associated with the primary and secondary endpoints could further help clinicians to identify patients at risk for early CNS disease progression (Table 3), survival (Table S1) and severe toxicity (Table S2). Taking these parameters into consideration, it is possible to make a more personalized treatment plan for every individual patient.

A limitation of this study was the retrospective nature of the partly prospective collected data, which could have led to loss of data. This detrimental effect of the design remains limited in our opinion, because the major events --CNS progression, PFS, OS, and severe toxicity-- have clinical consequences and were hence well-documented. Nevertheless, prospective validation must take place prior to implementation of these results. Another limitation is the lack of systematic imaging in patients without CNS metastases at baseline and during osimertinib treatment, because asymptomatic metastases or progression could have been missed. The risk of bias was low, because SNPs were genotyped after all data was collected. Furthermore, these results have not been validated externally, with an independent cohort. The here used alternative --internal validation by bootstrapping-- was considered to produce the most accurate validation results, and is statistically acceptable.^25^ The statistical robustness of our results is furthermore underlined by using a Bonferroni correction. Another limitation is selection bias, since the study cohort consisted of mainly Caucasian patients (91%). In the multivariate analyses, we did test and correct for ethnicity, but further studies should also focus on the genetic variance in other ethnic populations. Finally, we did not include the different resistance mechanisms, which could have confounded some results.

To conclude, *ABCG2* 34G>A and *ABCB1* 3435C>T are strong predictors for the development of new CNS metastases in patients treated with osimertinib for *EGFR*m+ NSCLC who have no documented CNS metastases. Furthermore, *ABCG2* 421C>A was significantly related with the incidence of severe osimertinib toxicity. These results encourage the use of pre-emptive genotyping these SNPs in clinical practice to further individualize osimertinib therapy, and point toward ABCB1 and ABCG2 as promising targets to prevent the development of new CNS metastases during osimertinib treatment.

## Contributors

G.D.M.V., A.C.D., and R.H.J.M. designed the study. G.D.M.V., R.J.B., M.J., A.J.v.d.W., I.B., L.E.L.H., S.C., C.M.J.S., E.d.J., S.L.W.K., N.S., R.H.N.v.S., E.F.S., A.C.D., and A.D.R.H. performed the research (*c.q.* inclusion of patients and data collection, including pharmacokinetic and pharmacogenetic data). G.D.M.V., E.O.-d.H., A.C.D. and R.H.J.M. analysed, verified, and interpreted the (raw) data. G.D.M.V. wrote the manuscript. All authors had full access to the data, critically reviewed the manuscript, and gave final approval for publication.

## Data sharing statement

All datasets including deidentified participant data, which are generated during and/or analysed during the current study are available from the corresponding author (g.veerman@erasmusmc.nl) on reasonable request until five years after date of publication. Data recipients are required to enter a formal data sharing agreement that describes the conditions for release and requirements for data transfer, storage, archiving, publication, and intellectual property with every participating centre.

The results of this study have been presented at the European Society of Medical Oncology congress (poster 27) in Paris, September 2022. The abstract has been awarded a merit award by the organizing committee.

## Declaration of interests

G.D.M.V. reports grants from Eli Lilly; outside the submitted work. A.C.D. reports grants from Boehringer-Ingelheim; outside the submitted work (paid to institution). R.H.J.M. reports an unrestricted grant from Boehringer-Ingelheim (paid to institution) and grants from Astellas, Bayer, Cristal Therapeutics, Novartis, Pamgene, Pfizer, Sanofi, and Servier; outside the submitted work (paid to institution). L.E.L.H. has no relationship to disclose in relation to this manuscript. Outside of the current manuscript: research funding Roche Genentech, AstraZeneca, Boehringer Ingelheim, Takeda (all institution, Beigene under negotiation); advisory board: BMS, Eli Lilly, Roche Genentech, Pfizer, Takeda, MSD, Merck, Novartis, Boehringer Ingelheim, Amgen, Janssen (all institution, Roche one time self); speaker: MSD, Lilly (institution); travel/conference reimbursement: Roche Genentech (self); mentorship program with key opinion leaders: funded by AstraZeneca; fees for educational webinars: Benecke, Medtalks, VJOncology (self), high5oncology (institution); interview sessions funded by Roche Genentech, Bayer, Lilly (institution); local PI of clinical trials: AstraZeneca, Novartis, BMS, MSD, Merck, GSK, Takeda, Blueprint Medicines, Roche Genentech, Janssen Pharmaceuticals, Mirati, Abbvie, Gilead. A.J.v.d.W. reports grants and advisory board from AstraZeneca, grants and advisory board from Boehringer-Ingelheim, advisory board from Janssen, advisory board from Novartis, grants and advisory board from Pfizer, grants and advisory board from Roche, grants and advisory board from Takeda, all outside the submitted work; all payment to the UMCG. N.S. provided consultation or attended advisory boards for Boehringer Ingelheim, Cogent Biosciences, Ellipses Pharma, Luszana. N. Steeghs received research grants from Abbvie, Actuate Therapeutics, Amgen, Array, Ascendis Pharma, AstraZeneca, Bayer, Blueprint Medicines, Boehringer Ingelheim, Bristol-Myers Squibb, Cantargia, CellCentric, Cogent Biosciences, Cresecendo Biologics, Cytovation, Deciphera, Eli Lilly, Exelixis, Genentech, GlaxoSmithKline, Incyte, InteRNA, Janssen, Kinnate Biopharma, Luszana, Merck, Merck Sharp & Dohme, Merus, Molecular Partners, Navire Pharma, Novartis, Numab Therapeutics, Pfizer, Relay Pharmaceuticals, Revolution Medicin, Roche, Sanofi, Seattle Genetics, Taiho, Takeda. All outside the submitted work, all payment to the Netherlands Cancer Institute. E.F.S. has no relationship to disclose in relation to the manuscript. Outside the submitted work (all institutional): research grants from Astra Zeneca, MSD, BMS, Roche Genentech, advisory boards Astra Zeneca, BMS, Boehringer Ingelheim, Bayer, DSI, MSD, Takeda, Roche, Merck, Novartis, Amgen, Janssen.

All other authors declare no competing interests.

## Appendix A Supplementary data

Supplementary Table S2

Supplementary Fig. 1 **Flowchart of all collected pharmacogenetic and pharmacokinetic samples.** The total study cohort consisted of samples from the NKI-AvL (N13FPB, IRBd19-192), Erasmus and Amphia (START-TKI, MEC 16-643; www.clinicaltrials.gov NCT05221372), MUMC (2019-1018-A-10 and OSIBOOST; www.clinicaltrials.gov NCT03858491), UMCG (Oncolifes biobank, OLS032-202000693), and AUMC (Liquid Biopsy Center biobank, UVB21-0125). Abbreviations: NKI-AvL = The Netherlands Cancer Institute – Antoni van Leeuwenhoek; Erasmus = Erasmus University Medical Centre; MUMC = Maastricht University Medical Centre; UMCG = University Medical Centre Groningen; AUMC = Amsterdam University Medical Centre; Amphia = Amphia hospital Breda; SNP = single nucleotide polymorphism; PK = pharmacokinetic.

Supplementary Fig. 2A **Central nervous system efficacy of osimertinib in patients without known brain metastases.** Figure A: Cumulative incidence of central nervous system metastases in patients which are divided based on *ABCG2* 34G>A. The blue line represents patients with *ABCG2* 34GG, the red line represents patients with *ABCG2* 34AG/AA. The 12-months incidence for the *ABCG2* 34GG cohort was 8.3%, compared to 1.9% in the *ABCG2* 34AG/AA cohort. Figure B: Cumulative incidence of central nervous system metastases in patients which are divided based on *ABCB1* 3435C>T. The blue line represents patients with *ABCB1* 3435CC, the red line represents patients with *ABCB1* 3435CT/TT. The 12-months incidence for the *ABCB1* 3435CC cohort was 3.1%, compared to 8.5% in the *ABCB1* 3435CT/TT cohort.

Supplementary Fig. 2B

Supplementary Fig. 3 **Incidence of severe osimertinib toxicity.** Cumulative incidence of severe toxicity in all patients treated with osimertinib, which are divided based on *ABCG2* 421C>A. The blue line represents patients with *ABCG2* 421CC, the red line represents patients with *ABCG2* 421CA/AA. The 12-months incidence for the *ABCG2* 421CC cohort was 15.1%, compared to 23.8% in the *ABCG2* 421CA/AA cohort.

Protocol
