# Supplementary material for: Influence of germline variations in drug transporters ABCB1 and ABCG2 on intracerebral osimertinib efficacy in patients with non-small cell lung cancer
Source: eClinicalMedicine. 2023 Apr 13;59:101955. doi: 10.1016/j.eclinm.2023.101955 (PMC10139887; doi:10.1016/j.eclinm.2023.101955)
Supplement: Supplementary Table S1 [file mmc1.docx]

**Table S1. Patient demographics and SNPs associated with progression-free survival and overall survival.**

|  | Progression-free survival in months (n=570) | | Overall survival in months (n=570) | |
| --- | --- | --- | --- | --- |
| Parameter | **Univariate median**  **(95% CI; p-value)** | **Multivariate HR**  **(95% CI; p-value)** | **Univariate median**  **(95% CI; p-value)** | **Multivariate HR**  **(95% CI)** |
| Sex  *Female vs male* | 10·7 vs 8·7; 9·11 - 12·27 vs 6·67 - 10·77 (0·042) | 0·752  (0·614 - 0·922; 0·006) | 26·0 vs 21·8; 23·67 - 28·33 vs 16·75 - 26·79 (0·136) |  |
| Age (years)  *<66 vs >65 year^1^* | 8·0 vs 11·6; 6·59 - 9·47 vs  9·82 - 13·40 (0·004) | 0·987  (0·979 - 0·996; 0·005) | 25·3 vs 25·0; 20·77 - 29·13 vs 22·55 - 27·96 (0·608) |  |
| Ethnicity  *Asian vs other* | 19·7 vs 9·7; 10·79 - 28·69 vs 8·48 - 11·00 (0·015) | 0·528  (0·360 - 0·774; 0·001) | 47·8 vs 24·3; 32·37 - 63·31 vs 22·25 - 26·27 (0·007) | 0·482  (0·285 - 0·817; 0·007) |
| BMI (in kg/m^2^)  *>22·9 vs <23^1^* | 11·7 vs 7·3; 10·21 - 13·01 vs 5·86 - 8·64 (0·007 | 0·973  (0·951 - 0·995; 0·018) | 27·0 vs 18·2; 24·45 - 29·59 vs 15·92 - 20·48 (<0·001) | 0·966  (0·939 - 0·994; 0·016) |
| WHO PS  *>1 vs 0-1* | 6·3 vs 10·8; 3·93 - 8·60 vs  9·25 - 12·25 (<0·001) | 1·466  (1·143 - 1·883; 0·003) | 15·8 vs 27·2; 11·84 - 19·84 vs 24·51 - 29·79 (<0·001) | 2·381  (1·779 - 3·185; <0·001) |
| Smoking  *Former/current vs never* | 9·7 vs 10·5; 7·84 - 11·64 vs 8·40 - 12·52 (0·229) |  | 24·0 vs 25·5; 21·10 - 26·84 vs 22·97 - 28·05 (0·318) |  |
| Primary EGFR mutation  *pL858R vs classic exon 19 del*  *Other vs classic exon 19 del* | 8·1 vs 12·6; 5·55 - 10·59 vs 10·88 - 14·36 (<0·001)  6·5 vs 12·6; 4·16 - 8·76 vs 10·88 - 14·36 (<0·001) | 1·473  (1·193 - 1·818; <0·001)  2·057  (1·541 - 2·746; <0·001) | 22·4 vs 28·7; 19·28 - 25·59 vs 24·26 - 33·12 (<0·001)  17·0 vs 28·7; 11·67 - 22·37 vs 24·26 - 33·12 (<0·001) | 1·482  (1·135 - 1·935; 0·004)  2·239  (1·595 - 3·143; <0·001) |
| Presence of TP53  *Yes vs no* | 8·0 vs 13·3; 6·87 - 9·14 vs 10·86 - 15·76 (<0·001) | 1·318  (1·006 - 1·727; 0·045) | 24·0 vs 30·0; 20·75 - 27·19 vs 22·62 - 37·32 (0·013) | 1·504  (1·082 - 2·092; 0·015) |
| Line of treatment  *First vs second* | 13·3 vs 9·1; 10·72 - 15·90 vs 7·74 - 10·42 (0·016) | 0·688  (0·550 - 0·860; 0·001) | 32·4 vs 24·0; 23·89 - 40·83 vs 21·70 - 26·24 (0·034) | 0·601  (0·439 – 0·822; 0·001) |
| Other prior treatment  *Yes vs no* | 8·0 vs 10·8; 6·31 - 9·75 vs  9·25 - 12·33 (0·047) |  | 21·3 vs 25·6; 16·14 - 26·42 vs 23·04 - 28·10 (0·032) |  |
| Baseline CNS metastases  *Yes vs no* | 7·4 vs 11·5; 6·37 - 8·51 vs 10·00 - 13·08 (<0·001) | 1·274  (1·042 – 1·555; 0·018) | 22·4 vs 27·9; 20·24 - 24·62 vs 23·86 - 32·00 (<0·001) | 1·309  (1·025 - 1·672; 0·031) |
| ABCB1 3435C>T dominant  *CT/TT vs CC* | 9·3 vs 10·8; 7·68 - 10·88 vs 8·97 - 12·67 (0·177) |  | 24·0 vs 30·9; 21·71 - 26·23 vs 16·21 - 45·49 (0·010) |  |
| ABCB1 3435C>T recessive  *TT vs CT/CC* | 10·7 vs 10·1; 7·78 - 13·60 vs 8·58 - 11·68 (0·265) |  | 24·8 vs 25·3; 19·92 - 29·72 vs 22·79 - 27·77 (0·792) |  |
| ABCG2 421C>A dominant  *CA/AA vs CC* | 9·7 vs 10·4; 6·81 - 12·68 vs 8·99 - 11·79 (0·692) |  | 25·5 vs 25·3; 20·20 - 30·89 vs 22·80 - 27·70 (0·805) |  |
| ABCG2 34G>A dominant  *GA/AA vs GG* | 11·5 vs 10·1; 8·40 - 14·56 vs 8·67 - 11·59 (0·948) |  | 27·3 vs 24·3; 21·69 - 32·99 vs 22·04 - 26·62 (0·951) |  |
| CYP3A4*22 dominant  *CT/TT vs CC* | 10·8 vs 10·2; 6·89 - 14·61 vs 8·58 - 11·55 (0·574) |  | 24·8 vs 25·3; 16·79 - 32·86 vs 23·08 - 27·48 (0·938) |  |

Association between patient demographics and SNPs with progression-free survival (PFS) and overall survival (OS).

Abbreviations: SNP = single nucleotide polymorphism; CNS = central nervous system; HR = hazard ratio; CI = confidence interval; BMI = body mass index; kg = kilograms; m = meter; WHO = World Health Organisation; EGFR = epidermal growth factor receptor; vs = versus. ^1^ subdivided in univariate analysis only.
