# Supplementary material for: Influence of germline variations in drug transporters ABCB1 and ABCG2 on intracerebral osimertinib efficacy in patients with non-small cell lung cancer
Source: eClinicalMedicine. 2023 Apr 13;59:101955. doi: 10.1016/j.eclinm.2023.101955 (PMC10139887; doi:10.1016/j.eclinm.2023.101955)
Supplement: Supplementary Table S2 [file mmc2.docx]

**Table S2. Patient demographics and SNPs associated with severe toxicity and pharmacokinetics.**

|  | Severe toxicity (n=572) | | | Pharmacokinetics (n=476)^2^ | |
| --- | --- | --- | --- | --- | --- |
| Parameter | **Univariate competing risk**  **HR (95% CI; p-value)** | **Multivariate competing risk HR (95% CI; p-value)** | **Incidence**  **(%; p-value Chi^2^)** | **Non-parametric p-value** | **Parametric mean ±SD; p-value (ng/ml)** |
| Sex  *Female vs male* | 1·674 (1·056 - 2·653; 0·028) | 1·754  (1·117 - 2·755; 0·015) |  |  |  |
| Age (years)  *<66 vs >65 year^1^* | 1·069 (1·044 - 1·095; <0·001) | 1·067  (1·042 - 1·093; <0·001) |  |  |  |
| Ethnicity  *Asian vs other* | 0·917 (0·430 - 1·959; 0·823) |  |  |  |  |
| BMI (in kg/m^2^)  *>22·9 vs <23^1^* | 0·990 (0·943 - 1·039; 0·683) |  |  |  |  |
| WHO PS  *>1 vs 0-1* | 1·189 (0·728 - 1·943; 0·489) |  |  |  |  |
| Smoking  *Former/current vs never* | 0·869 (0·594 - 1·271; 0·469) |  |  |  |  |
| Primary EGFR mutation  *pL858R vs classic exon 19 del*  *Other vs classic exon 19 del* | 1·156 (0·753 - 1·777; 0·508)  1·104 (0·613 - 1·990; 0·741) |  |  |  |  |
| Presence of TP53  *Yes vs no* | 0·711 (0·468 - 1·080; 0·223) |  |  |  |  |
| Line of treatment  *Second vs first* | 0·508 (0·346 - 0·747; 0·001) | 0·553  (0·378 - 0·808; 0·002) |  |  |  |
| Other prior treatment  *Yes vs no* | 0·643 (0·381 - 1·086; 0·098) |  |  |  |  |
| Baseline CNS metastases  *Yes vs no* | 0·623 (0·357 - 1·087; 0·240) |  |  |  |  |
| *ABCB1* 3435C>T dominant  *CT/TT vs CC* | 0·708 (0·468 - 1·071; 0·102) |  | 17·0 vs 23·4; 0·095 | 0·783 | 232 ±131 vs 242 ±91; 0·446 |
| *ABCB1* 3435C>T recessive  *TT vs CT/CC* | 1·169 (0·771 - 1·773; 0·461) |  | 20·3 vs 17·9; 0·520 | 0·036 | 215 ±83 vs 241 ±108; 0·007 |
| *ABCG2* 421C>A dominant  *CA/AA vs CC* | 1·687 (1·115 - 2·554; 0·013) |  | 27·0 vs 16·5; 0·010 | 0·647 | 231 ±97 vs 236 ±104; 0·672 |
| *ABCG2* 34G>A dominant  *GA/AA vs GG* | 0·823 (0·457 - 1·484; 0·518) |  | 15·7 vs 19·1; 0·453 | 0·205 | 243 ±89 vs 233 ±105; 0·459 |
| *CYP3A4*22* dominant  *CT/TT vs CC* | 1·108 (0·620 - 1·979; 0·730) |  | 20·0 vs 18·4; 0·757 | 0·400 | 251 ±144 vs 232 ±96; 0·209 |

Association between patient demographics and SNPs with severe toxicity and pharmacokinetics.

Severe toxicity was defined as Common Terminology Criteria for Adverse Events (CTCAE)(22) grade >2 adverse events, all toxicity which lead to dose reductions, treatment discontinuation or stop, and hospital admissions. Abbreviations: SNP = single nucleotide polymorphism; CNS = central nervous system; HR = hazard ratio; CI = confidence interval; BMI = body mass index; kg = kilograms; m = meter; WHO = World Health Organisation; EGFR = epidermal growth factor receptor; vs = versus. ^1^ subdivided in univariate analysis only; ^2^ for *ABCG2* 421C>A and *ACBG2* 34G>A 473 patients were included and for *CYP3A4*22* 474 patients were included in the analyses.
