# Supplementary figures and images for: Influence of germline variations in drug transporters ABCB1 and ABCG2 on intracerebral osimertinib efficacy in patients with non-small cell lung cancer
Source: eClinicalMedicine. 2023 Apr 13;59:101955. doi: 10.1016/j.eclinm.2023.101955 (PMC10139887; doi:10.1016/j.eclinm.2023.101955)

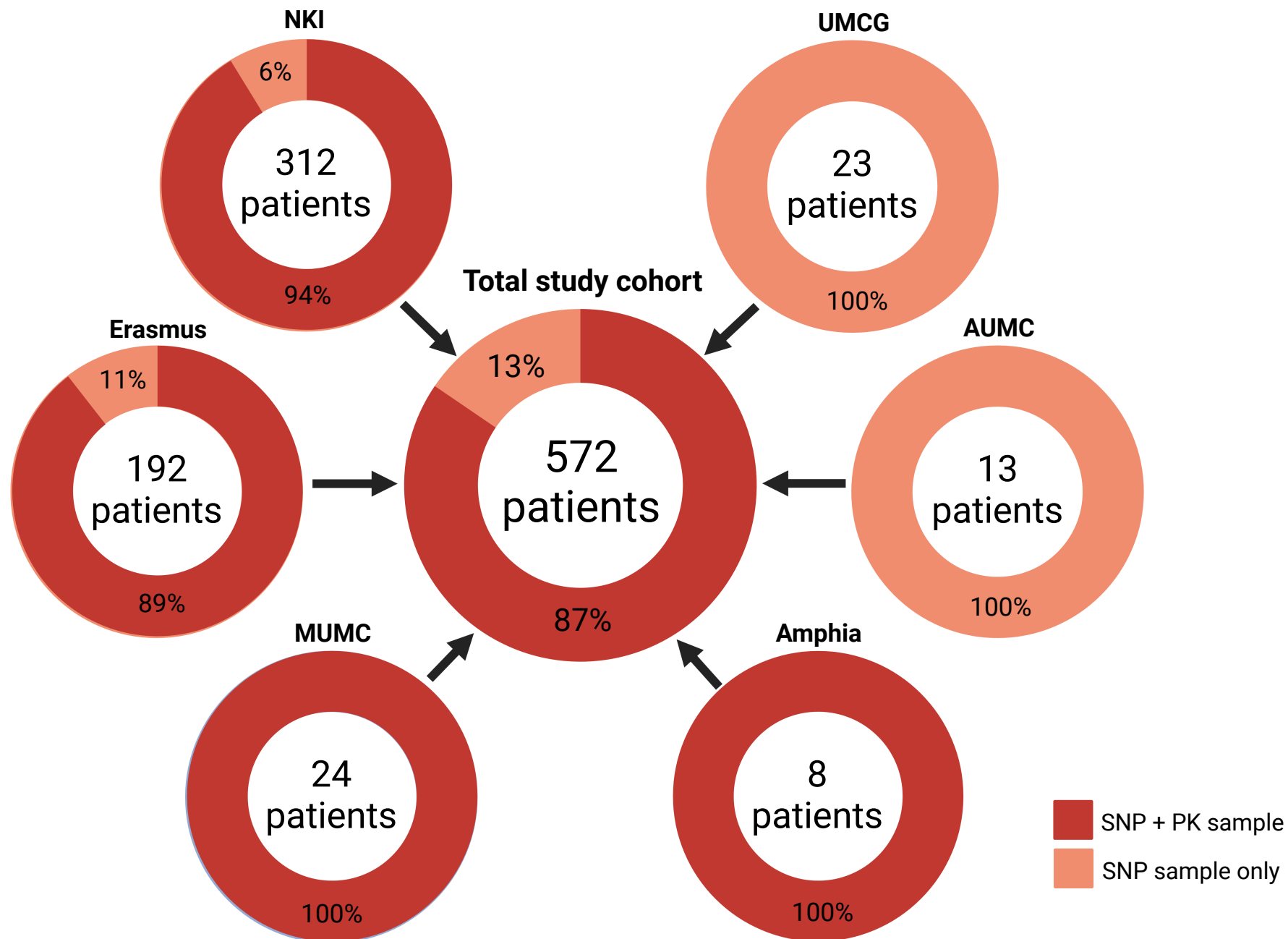

Supplement: Supplementary Fig. 1 — Flowchart of all collected pharmacogenetic and pharmacokinetic samples. The total study cohort consisted of samples from the NKI-AvL (N13FPB, IRBd19-192), Erasmus and Amphia (START-TKI, MEC 16-643; www.clinicaltrials.gov NCT05221372), MUMC (2019-1018-A-10 and OSIBOOST; www.clinicaltrials.gov NCT03858491), UMCG (Oncolifes biobank, OLS032-202000693), and AUMC (Liquid Biopsy Center biobank, UVB21-0125). Abbreviations: NKI-AvL = The Netherlands Cancer Institute – Antoni van Leeuwenhoek; Erasmus = Erasmus University Medical Centre; MUMC = Maastricht University Medical Centre; UMCG = University Medical Centre Groningen; AUMC = Amsterdam University Medical Centre; Amphia = Amphia hospital Breda; SNP = single nucleotide polymorphism; PK = pharmacokinetic. [file mmc3.pdf]

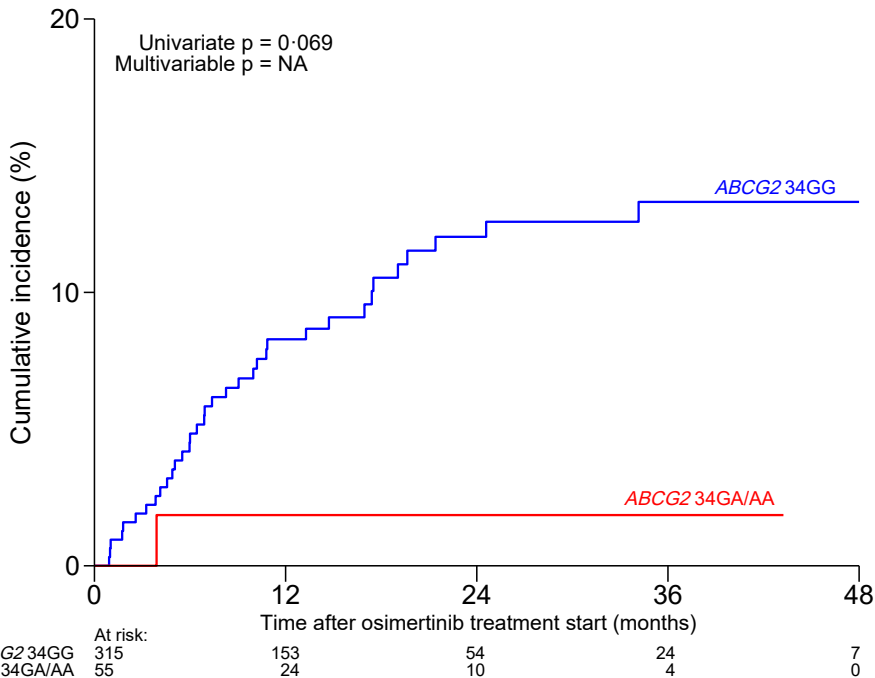

Supplement: Supplementary Fig. 2A — Central nervous system efficacy of osimertinib in patients without known brain metastases. Figure A: Cumulative incidence of central nervous system metastases in patients which are divided based on ABCG2 34G>A. The blue line represents patients with ABCG2 34GG, the red line represents patients with ABCG2 34AG/AA. The 12-months incidence for the ABCG2 34GG cohort was 8.3%, compared to 1.9% in the ABCG2 34AG/AA cohort. Figure B: Cumulative incidence of central nervous system metastases in patients which are divided based on ABCB1 3435C>T. The blue line represents patients with ABCB1 3435CC, the red line represents patients with ABCB1 3435CT/TT. The 12-months incidence for the ABCB1 3435CC cohort was 3.1%, compared to 8.5% in the ABCB1 3435CT/TT cohort. [file mmc4.pdf]

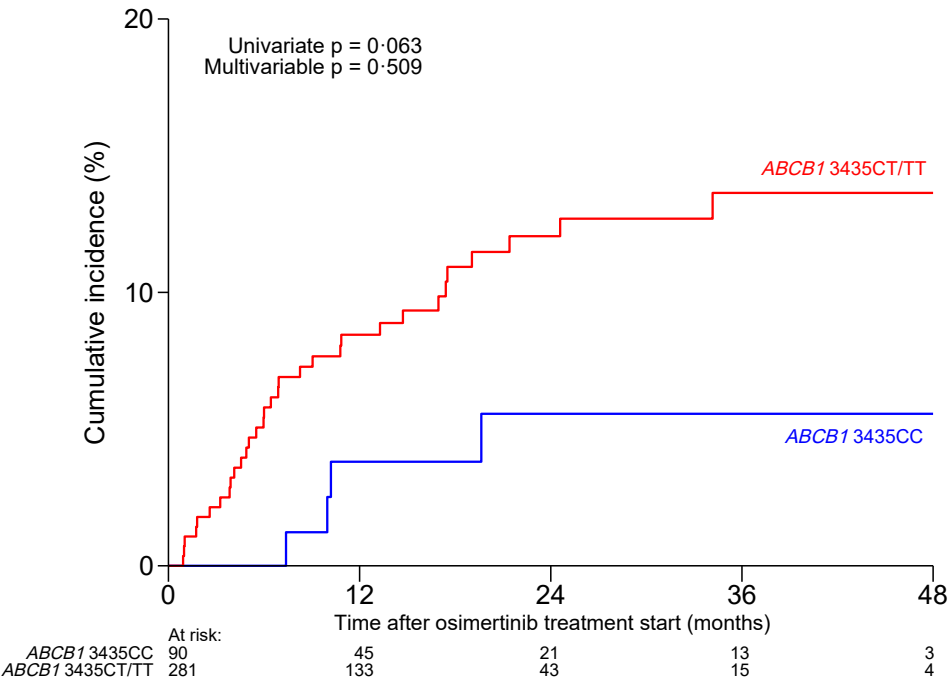

Supplement: Supplementary Fig. 2B [file mmc5.pdf]

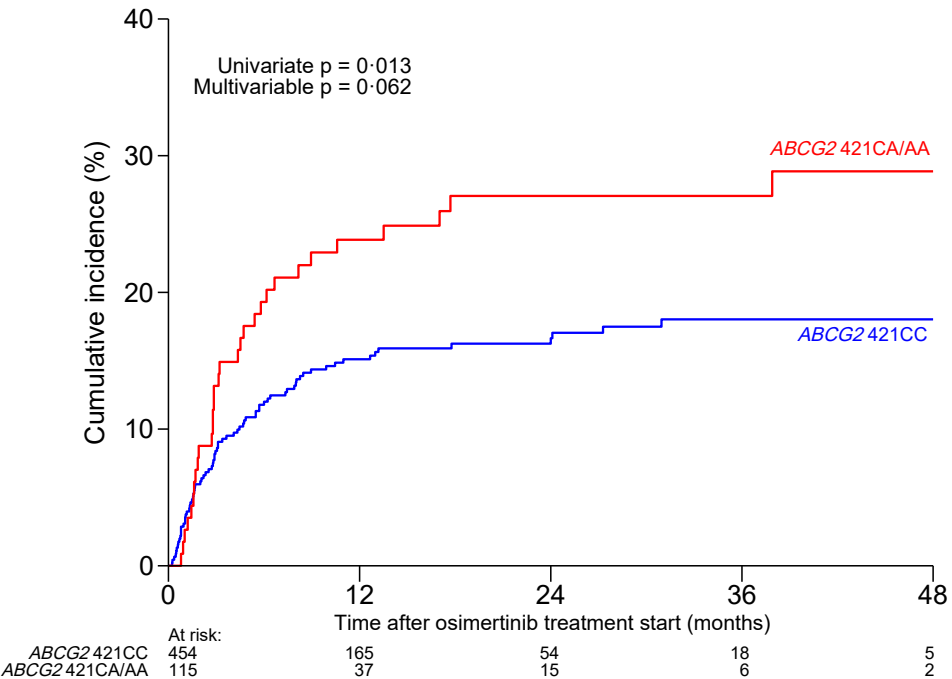

Supplement: Supplementary Fig. 3 — Incidence of severe osimertinib toxicity. Cumulative incidence of severe toxicity in all patients treated with osimertinib, which are divided based on ABCG2 421C>A. The blue line represents patients with ABCG2 421CC, the red line represents patients with ABCG2 421CA/AA. The 12-months incidence for the ABCG2 421CC cohort was 15.1%, compared to 23.8% in the ABCG2 421CA/AA cohort. [file mmc6.pdf]
