# Supplementary material for: Influence of germline variations in drug transporters ABCB1 and ABCG2 on intracerebral osimertinib efficacy in patients with non-small cell lung cancer
Source: eClinicalMedicine. 2023 Apr 13;59:101955. doi: 10.1016/j.eclinm.2023.101955 (PMC10139887; doi:10.1016/j.eclinm.2023.101955)
Supplement: Protocol [file mmc7.pdf]

Influence of the drug transporters ABCB1 and ABCG2 and enzyme CYP3A4 on survival outcome and pharmacokinetics in patients with non-small cell lung cancer treated with osimertinib; “the TRANSPORT study”

MEC 2020-557

Version 1: 17-06-2020

Writing committee

**G.D.M. Veerman, A.C. Dingemans, E. Oomen-de Hoop,
S.L.W. Koolen, R.H.N. van Schaik, R.H.J. Mathijssen**

Rationale
Brain metastases are frequently present in patients with lung cancer. Of all EGFR TKI's, drug penetration across the blood-brain barrier is highest for osimertinib.[1] Possibly therefor, the incidence of central nervous system (CNS) disease progression was lower for osimertinib compared to other EGFR TKI's.[2] This could suggest treatment efficacy to be correlated with CNS drug concentrations. Drug efflux transporters ABCB1 (P-glycoprotein; P-gP) and ABCG2 (breast cancer resistance protein; BCRP) are highly frequent present on the blood-brain barrier and synergize the efflux effect of various anti-cancer drugs, resulting in drug resistance.[3] Osimertinib is a substrate of P-glycoprotein and BCRP and is an inhibitor of BCRP, but does not inhibit P-glycoprotein.[4] Multiple single nucleotide polymorphisms (SNPs) are described which impair the function of both drug transporters. Most frequent described are the C3435T SNP in ABCB1 and the C421A and G34A SNP in ABCG2.[5-7] The influence of P-gP and BCRP on intracerebral osimertinib concentrations is unknown. Moreover, the effects of SNPs in these efflux transporters on treatment outcome (response and occurrence of CNS metastases) are not yet studied. Also effects of P-gP and BCRP on systemic osimertinib concentrations are unknown.
Osimertinib is mainly metabolized by CYP3A4.[4] The SNP CYP3A4*22 is associated with low hepatic CYP3A4 expression and CYP3A4 activity,[8] thus potentially increasing the systemic concentrations of osimertinib.

Study population
Patients who are or have been treated with osimertinib for non-small cell lung cancer (NSCLC) and of whom blood has been withdrawn for study or diagnostic purposes are eligible for usage in this study. There are multiple studies in which blood is or has been withdrawn from these specific patient population. Examples of these studies are, but are not limited to, the START-TKI study (MEC 16-643), the Code Geno study (MEC 02-1002), the MERLOT study (MEC 16-697), and BIOGIO study (MEC 17-251). When no clinical data is available for analysis, patients have to be excluded.

Power analysis
To have sufficient power in this study with three genes (four SNP's; prevalences shown in appendix A) and PK as parameters, at least 40-50 events have to take place for multivariate analysis in a discovery cohort. Based on CNS events, which is around 30% (appendix A), minimal number of to be included patients is 167. These numbers are idem ditto for a validation cohort. Hence, our aim is to include at least 500 patients.

Treatment of patients
No intervention is planned for this study nor will patients be informed of the outcomes of this study.

Methods - endpoints
Osimertinib treated patients will be divided in two groups, depending on the presence of CNS metastases at start of treatment.
In the group with CNS metastases at baseline, primary outcome is the correlation of the SNPs C3435T (ABCB1), C421A and G34A (ABCG2) and CYP3A4*22 with treatment response of CNS metastases. PFS will be measured as time to cerebral progressive disease or death from any cause. In the group without CNS metastases at baseline, primary endpoint is correlation between the four SNPs and de novo occurrence of CNS metastases (defined as time to brain metastasis).

Secondary endpoints are the correlations between presence of SNPs and OS, PFS independent of site (CNS or extracranial), toxicity, and pharmacokinetic parameters (AUC, Cmax, C/L) in the total cohort.

Methods - SNP analysis
Blood samples of patients treated with osimertinib will be analyzed for the SNPs C3435T (ABCB1), C421A and G34A (ABCG2) and CYP3A4*22. Analysis do not generate genetic information which will be leading to a specific patient nor will any outcome involve predicting changes for (future) diseases for the patient or his/her family.
Pharmacokinetics will be determined by quantifying the osimertinib concentration at steady state with time between last intake and blood withdrawal of > 6 hours.

Statistical analysis
Survival analysis with Kaplan-Meier and Cox regression for multi-variable analysis will be performed for the primary endpoints of both subgroups and secondary survival endpoints. Chi-square test will be performed for toxicity endpoints and Mann-Whitney U-tests for pharmacokinetics in a dominant model or Kruskal Wallis when P-gP is analyzed separately (CC vs CT vs TT) for the total cohort.

Appendix A: Prevalence of SNPs and CNS metastases

ABCB1 (P-gP): C3435T (CC 26-31%, CT 44-47%, TT 22-30%).

ABCG2 (BCRP): C421A (CC 74-86%, CA 14-26%, AA <1%) and G34A (GG 91%, AG 9%, AA <1%).

CYP3A4: CYP3A4*22 in 10%.

Osimertinib (data from the START-TKI study; MEC 16-643): at baseline, 29% had CNS metastases. At progressive disease, 30% had CNS progression.

References

[1] M.S. Ahluwalia, K. Becker, B.P. Levy, Epidermal Growth Factor Receptor Tyrosine Kinase Inhibitors for Central Nervous System Metastases from Non-Small Cell Lung Cancer, Oncologist, 23 (2018) 1199-1209.
[2] T. Reungwetwattana, K. Nakagawa, B.C. Cho, M. Cobo, E.K. Cho, A. Bertolini, S. Bohnet, C. Zhou, K.H. Lee, N. Nogami, I. Okamoto, N. Leighl, R. Hodge, A. McKeown, A.P. Brown, Y. Rukazenkov, S.S. Ramalingam, J. Vansteenkiste, CNS Response to Osimertinib Versus Standard Epidermal Growth Factor Receptor Tyrosine Kinase Inhibitors in Patients With Untreated EGFR-Mutated Advanced Non-Small-Cell Lung Cancer, J Clin Oncol, (2018) JCO2018783118.
[3] S. Agarwal, A.M. Hartz, W.F. Elmquist, B. Bauer, Breast cancer resistance protein and P-glycoprotein in brain cancer: two gatekeepers team up, Curr Pharm Des, 17 (2011) 2793-2802.
[4] F.D.A. U.S., Approval rapport TAGRISSO™ (osimertinib) Reference ID: 3846512, (Revised: 11/2015).
[5] J. Li, G. Cusatis, J. Brahmer, A. Sparreboom, R.W. Robey, S.E. Bates, M. Hidalgo, S.D. Baker, Association of variant ABCG2 and the pharmacokinetics of epidermal growth factor receptor tyrosine kinase inhibitors in cancer patients, Cancer Biol Ther, 6 (2007) 432-438.
[6] G. Cusatis, V. Gregorc, J. Li, A. Spreafico, R.G. Ingersoll, J. Verweij, V. Ludovini, E. Villa, M. Hidalgo, A. Sparreboom, S.D. Baker, Pharmacogenetics of ABCG2 and adverse reactions to gefitinib, J Natl Cancer Inst, 98 (2006) 1739-1742.
[7] F. Wang, Y.J. Liang, X.P. Wu, L.M. Chen, K.K. To, C.L. Dai, Y.Y. Yan, Y.S. Wang, X.Z. Tong, L.W. Fu, Prognostic value of the multidrug resistance transporter ABCG2 gene polymorphisms in Chinese patients with de novo acute leukaemia, Eur J Cancer, 47 (2011) 1990-1999.
[8] L. Elens, T. van Gelder, D.A. Hesselink, V. Haufroid, R.H. van Schaik, CYP3A4*22: promising newly identified CYP3A4 variant allele for personalizing pharmacotherapy, Pharmacogenomics, 14 (2013) 47-62.
